# 7-Hy­droxy­methyl-2-pivaloyl­amino-1,8-naphthyridine

**DOI:** 10.1107/S1600536813005527

**Published:** 2013-03-06

**Authors:** Hoong-Kun Fun, Ching Kheng Quah, Krishnendu Aich, Sangita Das, Shyamaprosad Goswami

**Affiliations:** a X-ray Crystallography Unit, School of Physics, Universiti Sains Malaysia, 11800 USM, Penang, Malaysia; b Department of Pharmaceutical Chemistry, College of Pharmacy, King Saud University, PO Box 2457, Riyadh 11451, Saudi Arabia; c Department of Chemistry, Bengal Engineering and Science University, Shibpur, Howrah 711 103, India

## Abstract

In the title compound, C_14_H_17_N_3_O_2_, the mean plane of the 1,8-naphthyridine ring system (r.m.s deviation = 0.020 Å) forms a dihedral angle of 23.4 (1)° with the acetamide moiety (r.m.s deviation = 0.001 Å). The mol­ecular structure is stabilized by an intra­molecular O—H⋯N hydrogen bond, which generates an *S*(5) ring motif. In the crystal, mol­ecules are linked into inversion dimers by pairs of N—H⋯O hydrogen bonds, generating 18-membered *R*_2_^2^(18) ring motifs.

## Related literature

For general background to and the medicinal properties of 1,8-naphthyridine derivatives see: Badawneh *et al.* (2001 ▶); Litvinov (2004 ▶). For standard bond-length data, see: Allen *et al.* (1987 ▶). For hydrogen-bond motifs, see: Bernstein *et al.* (1995 ▶).
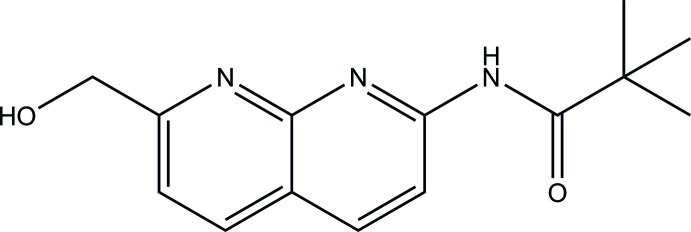


## Experimental

### 

#### Crystal data

C_14_H_17_N_3_O_2_
*M* *_r_* = 259.31
Monoclinic, 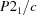

*a* = 14.7026 (3) Å
*b* = 6.2586 (1) Å
*c* = 14.7035 (3) Å
β = 97.447 (2)°
*V* = 1341.57 (4) Å^3^
*Z* = 4
Mo *K*α radiation
μ = 0.09 mm^−1^
*T* = 297 K
0.51 × 0.46 × 0.08 mm

#### Data collection

Bruker SMART APEXII CCD area-detector diffractometer
Absorption correction: multi-scan (*SADABS*; Bruker, 2009 ▶) *T*_min_ = 0.956, *T*_max_ = 0.993
20410 measured reflections
3949 independent reflections
2551 reflections with *I* > 2σ(*I*)
*R*_int_ = 0.042

#### Refinement

*R*[*F* ^2^ > 2σ(*F* ^2^)] = 0.074
*wR*(*F* ^2^) = 0.148
*S* = 1.14
3949 reflections
183 parameters
H atoms treated by a mixture of independent and constrained refinement
Δρ_max_ = 0.21 e Å^−3^
Δρ_min_ = −0.14 e Å^−3^

### 

Data collection: *APEX2* (Bruker, 2009 ▶); cell refinement: *SAINT* (Bruker, 2009 ▶); data reduction: *SAINT*; program(s) used to solve structure: *SHELXTL* (Sheldrick, 2008 ▶); program(s) used to refine structure: *SHELXTL*; molecular graphics: *SHELXTL*; software used to prepare material for publication: *SHELXTL* and *PLATON* (Spek, 2009 ▶).

## Supplementary Material

Crystal structure: contains datablock(s) global, I. DOI: 10.1107/S1600536813005527/rz5047sup1.cif

Structure factors: contains datablock(s) I. DOI: 10.1107/S1600536813005527/rz5047Isup2.hkl

Supplementary material file. DOI: 10.1107/S1600536813005527/rz5047Isup3.cml

Additional supplementary materials:  crystallographic information; 3D view; checkCIF report

## Figures and Tables

**Table 1:** Hydrogen-bond geometry (Å, °)

*D*—H⋯*A*	*D*—H	H⋯*A*	*D*⋯*A*	*D*—H⋯*A*
N3—H1*N*3⋯O2^i^	0.83 (2)	2.09 (2)	2.900 (2)	168 (2)
O2—H1*O*2⋯N2	0.86 (3)	2.10 (3)	2.648 (2)	121 (2)

Symmetry code: (i) 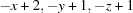.

**Fig. 1.**
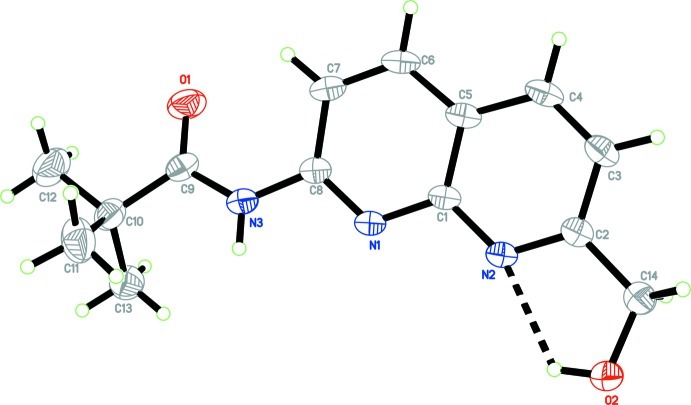
The molecular structure of the title compound showing 30% probability displacement ellipsoids for non-H atoms. The intramolecular hydrogen bond is shown as a dashed line.

**Fig. 2.**
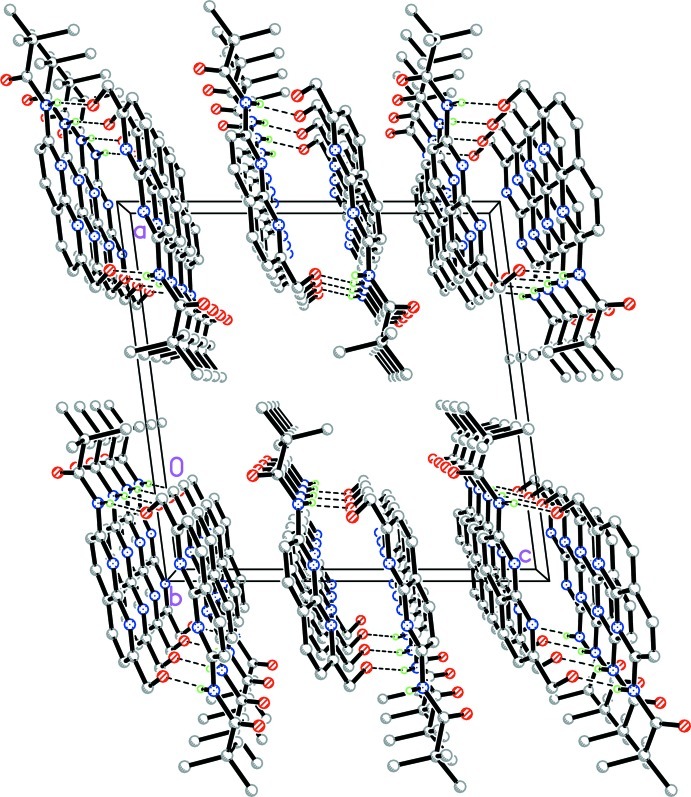
The crystal structure of the title compound, viewed along the b axis. H atoms not involved in hydrogen bonds (dashed lines) have been omitted for clarity.

## supplementary crystallographic information

### Comment

1,8-Naphthyridine system is of great interest due to their broad application in medicine as they are potentially useful as antihypertensives, antitumor agents, immunostimulants, and herbicide safeners (Badawneh *et al.*, 2001; Litvinov, 2004). Herein, we report the crystal structure of 2-pivaloylamino-7-hydroxymethyl-[1,8]naphthyridine.

In the title molecule, Fig. 1, the mean plane of [1,8]naphthyridine ring system (N1/N2/C1-C8, r.m.s deviation = 0.020 Å) forms a dihedral angle of 23.4 (1)° with the acetamide moiety (O1/N3/C9/C10, r.m.s deviation = 0.001 Å). Bond lengths (Allen *et al.*, 1987) and angles are within normal ranges. The molecular structure is stabilized by an intramolecular O2–H1O2···N2 (Table 1) hydrogen bond, which generates an S(5) ring motif (Bernstein *et al.*, 1995).

In the crystal, Fig. 2, molecules are linked into inversion dimers by pairs of intermolecular N3–H1N3···O2 (Table 1) hydrogen bonds, generating eighteen-membered R^2^_2_(18) ring motifs (Bernstein *et al.*, 1995).

### Experimental

To a stirred solution of 7-pivaloylamino-[1,8]naphthyridine-2-carbaldehyde (514 mg, 2 mmol) in dry THF was added NaBH_4_ (40 mg, 1mmol) at 0°C and the resulting mixture was stirred for half an hour at room temperature under nitrogen atmosphere. After evaporation of the solvent, water was added to it and then the reaction mixture was extracted with dichloromethane thrice. The organic layer was dried over anhydrous sodium sulphate and then evaporated under reduced pressure. The crude solid was purified through column chromatography (silica gel, 60-120 mesh) using ethyl acetate as eluent to afford a pure brown crystalline solid. Yield: 87%. M.p. 135-137 °C

### Refinement

Atoms H1N3 and H1O2 were located in a difference Fourier map and refined freely [N–H = 0.83 (2) Å and O–H 0.86 (3) Å]. The remaining H atoms were positioned geometrically and refined using a riding model with C–H = 0.93-0.97 Å and *U*_iso_(H) = 1.2 or 1.5 *U*_eq_(C). A rotating-group model was applied for the methyl groups.

### Crystal data

**Table:**

C_14_H_17_N_3_O_2_	*F*(000) = 552
*M**_r_* = 259.31	*D*_x_ = 1.284 Mg m^−^^3^
Monoclinic, *P*2_1_/*c*	Mo *K*α radiation, λ = 0.71073 Å
Hall symbol: -P 2ybc	Cell parameters from 4518 reflections
*a* = 14.7026 (3) Å	θ = 2.8–30.1°
*b* = 6.2586 (1) Å	µ = 0.09 mm^−^^1^
*c* = 14.7035 (3) Å	*T* = 297 K
β = 97.447 (2)°	Plate, brown
*V* = 1341.57 (4) Å^3^	0.51 × 0.46 × 0.08 mm
*Z* = 4	

### Data collection

**Table:**

Bruker SMART APEXII CCD area-detector diffractometer	3949 independent reflections
Radiation source: fine-focus sealed tube	2551 reflections with *I* > 2σ(*I*)
Graphite monochromator	*R*_int_ = 0.042
φ and ω scans	θ_max_ = 30.2°, θ_min_ = 2.8°
Absorption correction: multi-scan (*SADABS*; Bruker, 2009)	*h* = −20→20
*T*_min_ = 0.956, *T*_max_ = 0.993	*k* = −8→8
20410 measured reflections	*l* = −20→19

### Refinement

**Table:**

Refinement on *F*^2^	Primary atom site location: structure-invariant direct methods
Least-squares matrix: full	Secondary atom site location: difference Fourier map
*R*[*F*^2^ > 2σ(*F*^2^)] = 0.074	Hydrogen site location: inferred from neighbouring sites
*wR*(*F*^2^) = 0.148	H atoms treated by a mixture of independent and constrained refinement
*S* = 1.14	*w* = 1/[σ^2^(*F*_o_^2^) + (0.0366*P*)^2^ + 0.506*P*] where *P* = (*F*_o_^2^ + 2*F*_c_^2^)/3
3949 reflections	(Δ/σ)_max_ = 0.001
183 parameters	Δρ_max_ = 0.21 e Å^−^^3^
0 restraints	Δρ_min_ = −0.14 e Å^−^^3^

### Special details

**Table:**

Geometry. All esds (except the esd in the dihedral angle between two l.s. planes) are estimated using the full covariance matrix. The cell esds are taken into account individually in the estimation of esds in distances, angles and torsion angles; correlations between esds in cell parameters are only used when they are defined by crystal symmetry. An approximate (isotropic) treatment of cell esds is used for estimating esds involving l.s. planes.
Refinement. Refinement of F^2^ against ALL reflections. The weighted R-factor wR and goodness of fit S are based on F^2^, conventional R-factors R are based on F, with F set to zero for negative F^2^. The threshold expression of F^2^ > 2sigma(F^2^) is used only for calculating R-factors(gt) etc. and is not relevant to the choice of reflections for refinement. R-factors based on F^2^ are statistically about twice as large as those based on F, and R- factors based on ALL data will be even larger.

### Fractional atomic coordinates and isotropic or equivalent isotropic displacement parameters (Å^2^)

**Table:**

	*x*	*y*	*z*	*U*_iso_*/*U*_eq_	
O1	0.70708 (11)	−0.0049 (3)	0.72243 (11)	0.0731 (5)	
O2	1.20193 (11)	0.4754 (2)	0.50680 (10)	0.0564 (4)	
N1	0.93270 (10)	0.1958 (2)	0.60449 (9)	0.0398 (4)	
N2	1.08422 (10)	0.2310 (2)	0.58087 (9)	0.0403 (4)	
C1	1.01722 (12)	0.1059 (3)	0.60905 (10)	0.0369 (4)	
C2	1.16763 (13)	0.1520 (3)	0.58648 (11)	0.0424 (4)	
C3	1.19173 (15)	−0.0552 (3)	0.61930 (12)	0.0509 (5)	
H3A	1.2519	−0.1032	0.6226	0.061*	
C4	1.12550 (14)	−0.1827 (3)	0.64593 (12)	0.0495 (5)	
H4A	1.1396	−0.3206	0.6668	0.059*	
C5	1.03554 (13)	−0.1052 (3)	0.64177 (11)	0.0410 (4)	
C6	0.96182 (14)	−0.2230 (3)	0.66823 (11)	0.0468 (5)	
H6A	0.9707	−0.3639	0.6876	0.056*	
C7	0.87830 (14)	−0.1325 (3)	0.66566 (12)	0.0475 (5)	
H7A	0.8296	−0.2073	0.6850	0.057*	
C8	0.86672 (13)	0.0803 (3)	0.63253 (11)	0.0406 (4)	
N3	0.78116 (12)	0.1817 (3)	0.62256 (12)	0.0490 (4)	
C9	0.70570 (14)	0.1334 (3)	0.66391 (14)	0.0505 (5)	
C10	0.61984 (14)	0.2648 (3)	0.63154 (15)	0.0578 (6)	
C11	0.59481 (17)	0.2364 (5)	0.52733 (18)	0.0814 (8)	
H11A	0.5892	0.0869	0.5130	0.122*	
H11B	0.6420	0.2979	0.4962	0.122*	
H11C	0.5376	0.3066	0.5077	0.122*	
C12	0.54109 (18)	0.1846 (5)	0.6811 (2)	0.0986 (10)	
H12A	0.5286	0.0376	0.6656	0.148*	
H12B	0.4872	0.2687	0.6627	0.148*	
H12C	0.5580	0.1973	0.7462	0.148*	
C13	0.63721 (16)	0.5019 (4)	0.65370 (17)	0.0688 (6)	
H13A	0.6540	0.5190	0.7186	0.103*	
H13B	0.5824	0.5822	0.6344	0.103*	
H13C	0.6860	0.5530	0.6220	0.103*	
C14	1.23927 (14)	0.2947 (3)	0.55470 (14)	0.0536 (5)	
H14A	1.2810	0.3412	0.6075	0.064*	
H14B	1.2745	0.2136	0.5152	0.064*	
H1N3	0.7793 (14)	0.288 (4)	0.5889 (14)	0.057 (6)*	
H1O2	1.1439 (18)	0.474 (4)	0.5097 (18)	0.087 (9)*	

### Atomic displacement parameters (Å^2^)

**Table:**

	*U*^11^	*U*^22^	*U*^33^	*U*^12^	*U*^13^	*U*^23^
O1	0.0823 (11)	0.0626 (10)	0.0797 (11)	−0.0054 (9)	0.0311 (9)	0.0198 (9)
O2	0.0555 (9)	0.0436 (8)	0.0725 (10)	0.0026 (7)	0.0170 (8)	0.0102 (7)
N1	0.0502 (9)	0.0312 (8)	0.0379 (7)	0.0001 (7)	0.0052 (6)	0.0041 (6)
N2	0.0505 (9)	0.0323 (8)	0.0381 (7)	0.0023 (7)	0.0061 (6)	0.0028 (6)
C1	0.0543 (11)	0.0285 (9)	0.0277 (8)	0.0023 (8)	0.0041 (7)	−0.0003 (7)
C2	0.0531 (11)	0.0408 (10)	0.0333 (8)	0.0059 (9)	0.0058 (8)	−0.0014 (7)
C3	0.0610 (13)	0.0464 (12)	0.0453 (10)	0.0178 (10)	0.0074 (9)	0.0020 (9)
C4	0.0760 (14)	0.0356 (10)	0.0373 (9)	0.0169 (10)	0.0082 (9)	0.0056 (8)
C5	0.0648 (12)	0.0305 (9)	0.0276 (8)	0.0050 (8)	0.0060 (8)	−0.0004 (7)
C6	0.0770 (14)	0.0275 (9)	0.0354 (9)	0.0005 (9)	0.0058 (9)	0.0041 (7)
C7	0.0673 (13)	0.0343 (10)	0.0415 (10)	−0.0090 (9)	0.0087 (9)	0.0036 (8)
C8	0.0524 (11)	0.0351 (9)	0.0339 (8)	−0.0032 (8)	0.0035 (8)	−0.0002 (7)
N3	0.0524 (10)	0.0409 (9)	0.0547 (10)	−0.0029 (8)	0.0105 (8)	0.0112 (8)
C9	0.0579 (12)	0.0413 (11)	0.0538 (11)	−0.0123 (9)	0.0132 (9)	−0.0048 (9)
C10	0.0519 (12)	0.0484 (12)	0.0749 (14)	−0.0070 (10)	0.0151 (11)	−0.0055 (11)
C11	0.0617 (15)	0.0837 (19)	0.0930 (18)	0.0035 (13)	−0.0122 (13)	−0.0208 (15)
C12	0.0694 (17)	0.084 (2)	0.152 (3)	−0.0073 (15)	0.0491 (18)	0.009 (2)
C13	0.0699 (15)	0.0540 (14)	0.0838 (16)	−0.0001 (12)	0.0141 (12)	−0.0100 (12)
C14	0.0532 (12)	0.0512 (12)	0.0571 (12)	0.0051 (10)	0.0094 (9)	0.0030 (10)

### Geometric parameters (Å, º)

**Table:**

O1—C9	1.219 (2)	C8—N3	1.399 (2)
O2—C14	1.406 (2)	N3—C9	1.366 (2)
O2—H1O2	0.86 (3)	N3—H1N3	0.83 (2)
N1—C8	1.318 (2)	C9—C10	1.530 (3)
N1—C1	1.358 (2)	C10—C12	1.531 (3)
N2—C2	1.315 (2)	C10—C13	1.534 (3)
N2—C1	1.364 (2)	C10—C11	1.539 (3)
C1—C5	1.420 (2)	C11—H11A	0.9600
C2—C3	1.412 (3)	C11—H11B	0.9600
C2—C14	1.501 (3)	C11—H11C	0.9600
C3—C4	1.356 (3)	C12—H12A	0.9600
C3—H3A	0.9300	C12—H12B	0.9600
C4—C5	1.403 (3)	C12—H12C	0.9600
C4—H4A	0.9300	C13—H13A	0.9600
C5—C6	1.407 (3)	C13—H13B	0.9600
C6—C7	1.348 (3)	C13—H13C	0.9600
C6—H6A	0.9300	C14—H14A	0.9700
C7—C8	1.421 (3)	C14—H14B	0.9700
C7—H7A	0.9300		
			
C14—O2—H1O2	106.9 (18)	N3—C9—C10	115.32 (18)
C8—N1—C1	117.60 (15)	C9—C10—C12	108.7 (2)
C2—N2—C1	117.98 (15)	C9—C10—C13	110.25 (18)
N1—C1—N2	116.06 (15)	C12—C10—C13	109.4 (2)
N1—C1—C5	122.31 (17)	C9—C10—C11	109.20 (18)
N2—C1—C5	121.63 (16)	C12—C10—C11	109.8 (2)
N2—C2—C3	123.87 (18)	C13—C10—C11	109.6 (2)
N2—C2—C14	116.29 (16)	C10—C11—H11A	109.5
C3—C2—C14	119.83 (17)	C10—C11—H11B	109.5
C4—C3—C2	118.79 (18)	H11A—C11—H11B	109.5
C4—C3—H3A	120.6	C10—C11—H11C	109.5
C2—C3—H3A	120.6	H11A—C11—H11C	109.5
C3—C4—C5	119.48 (17)	H11B—C11—H11C	109.5
C3—C4—H4A	120.3	C10—C12—H12A	109.5
C5—C4—H4A	120.3	C10—C12—H12B	109.5
C4—C5—C6	124.22 (17)	H12A—C12—H12B	109.5
C4—C5—C1	118.24 (17)	C10—C12—H12C	109.5
C6—C5—C1	117.54 (17)	H12A—C12—H12C	109.5
C7—C6—C5	120.21 (17)	H12B—C12—H12C	109.5
C7—C6—H6A	119.9	C10—C13—H13A	109.5
C5—C6—H6A	119.9	C10—C13—H13B	109.5
C6—C7—C8	118.16 (18)	H13A—C13—H13B	109.5
C6—C7—H7A	120.9	C10—C13—H13C	109.5
C8—C7—H7A	120.9	H13A—C13—H13C	109.5
N1—C8—N3	113.99 (16)	H13B—C13—H13C	109.5
N1—C8—C7	124.12 (18)	O2—C14—C2	112.97 (16)
N3—C8—C7	121.81 (18)	O2—C14—H14A	109.0
C9—N3—C8	128.60 (18)	C2—C14—H14A	109.0
C9—N3—H1N3	118.5 (15)	O2—C14—H14B	109.0
C8—N3—H1N3	112.8 (15)	C2—C14—H14B	109.0
O1—C9—N3	122.1 (2)	H14A—C14—H14B	107.8
O1—C9—C10	122.61 (19)		
			
C8—N1—C1—N2	−178.99 (14)	C5—C6—C7—C8	2.3 (3)
C8—N1—C1—C5	0.6 (2)	C1—N1—C8—N3	−177.86 (14)
C2—N2—C1—N1	178.24 (14)	C1—N1—C8—C7	−1.0 (2)
C2—N2—C1—C5	−1.3 (2)	C6—C7—C8—N1	−0.4 (3)
C1—N2—C2—C3	0.5 (2)	C6—C7—C8—N3	176.20 (16)
C1—N2—C2—C14	179.94 (15)	N1—C8—N3—C9	−162.20 (18)
N2—C2—C3—C4	0.7 (3)	C7—C8—N3—C9	20.9 (3)
C14—C2—C3—C4	−178.71 (17)	C8—N3—C9—O1	5.0 (3)
C2—C3—C4—C5	−1.1 (3)	C8—N3—C9—C10	−174.93 (18)
C3—C4—C5—C6	−179.53 (17)	O1—C9—C10—C12	−2.5 (3)
C3—C4—C5—C1	0.3 (2)	N3—C9—C10—C12	177.52 (19)
N1—C1—C5—C4	−178.57 (15)	O1—C9—C10—C13	117.4 (2)
N2—C1—C5—C4	0.9 (2)	N3—C9—C10—C13	−62.6 (2)
N1—C1—C5—C6	1.3 (2)	O1—C9—C10—C11	−122.2 (2)
N2—C1—C5—C6	−179.22 (15)	N3—C9—C10—C11	57.8 (2)
C4—C5—C6—C7	177.13 (17)	N2—C2—C14—O2	−12.0 (2)
C1—C5—C6—C7	−2.7 (2)	C3—C2—C14—O2	167.53 (16)

### Hydrogen-bond geometry (Å, º)

**Table:**

*D*—H···*A*	*D*—H	H···*A*	*D*···*A*	*D*—H···*A*
N3—H1*N*3···O2^i^	0.83 (2)	2.09 (2)	2.900 (2)	168 (2)
O2—H1*O*2···N2	0.86 (3)	2.10 (3)	2.648 (2)	121 (2)

Symmetry code: (i) −*x*+2, −*y*+1, −*z*+1.
